# Litter Size Reduction as a Model of Overfeeding during Lactation and Its Consequences for the Development of Metabolic Diseases in the Offspring

**DOI:** 10.3390/nu14102045

**Published:** 2022-05-13

**Authors:** Luana L. Souza, Egberto G. Moura, Patricia C. Lisboa

**Affiliations:** Laboratory of Endocrine Physiology, Department of Physiological Sciences, Roberto Alcantara Gomes Biology Institute, State University of Rio de Janeiro, Rio de Janeiro 20551-031, RJ, Brazil; luana.lsouzaf@gmail.com (L.L.S.); egmoura3@gmail.com (E.G.M.)

**Keywords:** overnutrition, small litters, rat, mouse, obesity, DOHaD concept

## Abstract

Overfeeding during lactation has a deleterious impact on the baby’s health throughout life. In humans, early overnutrition has been associated with higher susceptibility to obesity and metabolic disorders in childhood and adulthood. In rodents, using a rodent litter size reduction model (small litter) to mimic early overfeeding, the same metabolic profile has been described. Therefore, the rodent small litter model is an efficient tool to investigate the adaptive mechanisms involved in obesogenesis. Besides central and metabolic dysfunctions, studies have pointed to the contribution of the endocrine system to the small litter phenotype. Hormones, especially leptin, insulin, and adrenal hormones, have been associated with satiety, glucose homeostasis, and adipogenesis, while hypothyroidism impairs energy metabolism, favoring obesity. Behavioral modifications, hepatic metabolism changes, and reproductive dysfunctions have also been reported. In this review, we update these findings, highlighting the interaction of early nutrition and the adaptive features of the endocrine system. We also report the sex-related differences and epigenetic mechanisms. This model highlights the intense plasticity during lactation triggering many adaptive responses, which are the basis of the developmental origins of health and disease (DOHaD) concept. Our review demonstrates the complexity of the adaptive mechanisms involved in the obesity phenotype promoted by early overnutrition, reinforcing the necessity of adequate nutritional habits during lactation.

## 1. Introduction

### Child Obesity and Metabolic Programming

The global prevalence of obesity and its comorbidities during childhood has increased worldwide [1]. In a recent systematic review, the global prevalence of metabolic syndrome was estimated to be approximately 2.8%, which corresponds to approximately 25.8 million children around the world [2]. In 2015, China, India, Brazil, and the USA showed the largest absolute number of obese boys aged 2–4 years [1].

Early obesity is a global concern since there is a strong correlation between childhood body weight and adult obesity and metabolic syndrome [3]. This could be associated with rapid weight gain (RWG) during the first two years of life, which promotes a 3.66 times greater odds of overweight/obesity development later in life (from 2 to 46.5 years) [3]. Many factors, such as low weight birth, could be involved [4], but early postnatal overfeeding, by incorrect use of infant formula, also acts as a risk factor for early RWG and later obesity [3,5]. Early weaned children (<4 months) exhibited early rapid growth until 3 years of age, which was associated with increased adiposity and overweight risk until 14 years of age [6]. Indeed, the energy intake at four months was associated with higher weight gain from birth to one, two, and three years old [5], which has been described as an important predictor of later obesity [3,7].

The Developmental Origins of Health and Disease (DOHaD) concept explains the correlation between the early life environment and increased susceptibility to diseases, such as obesity and metabolic syndrome, in adulthood [8]. Neonatal life, including lactation, is a period of increased plasticity and maturation of many organs and systems [9]. Therefore, postnatal under- or overnutrition promotes adaptive modifications by epigenetic mechanisms, adjusting the organism during this period of developmental plasticity to a future predicted environment, expressed as the hypothesis of mismatch as a potential cause of future diseases [10]. This inadequate prediction and associated metabolic adjustments may favor the offspring’s development of obesity, diabetes, dyslipidemia, hypertension, and other comorbidities in adulthood [8]. This phenomenon is termed developmental or metabolic programming and can explain the individual susceptibility to obesity and its comorbidities.

Many epidemiological and experimental studies have supported the DOHaD concept. These observations have increased the attention paid to the “The First Thousand Days of life” [11], which includes the immediate postconception period until 2 years old. Thus, it is necessary for health policies to emphasize the importance of adequate nutrition and healthier habits of both mother and child to prevent later obesity and chronic diseases.

In this review, we explore the recent findings of adaptive mechanisms in the small litter (SL) model responsible for metabolic disorders in the offspring. In view of the importance of the endocrine system in metabolic control, we emphasize hormonal changes, especially changes in its specific tissue action. We also explored the potential epigenetic mechanisms involved in this adaptive phenotype of the SL model and the interference of sex dimorphism with the metabolic profile.

## 2. The Litter Size Reduction Model: Short- and Long-Term Effects

### 2.1. Metabolic Syndrome

Early postnatal overfeeding, promoting higher susceptibility to obesity and its metabolic disorders throughout life, is experimentally simulated in rodents by the litter size reduction model [12,13,14]. In this model, in early life (3 or 4 days after birth), the litter is reduced to 3 or 4 pups per dam, while the control litter is maintained with 8 to 12 pups per dam [15]. Normally, rodent dams have fewer nipples than pups, which causes them to compete for suckling. Usually, males have more access than females to nipples, and some nipples produce less milk than others [16]. Thus, in the litter size reduction model, as the number of dam nipples is higher than the pup number, there is no competition for suckling. Therefore, a small litter (SL) exhibits a higher milk intake per pup (approximately 1.5-fold) than a normal-sized litter [17]. Additionally, the milk composition of SL dams changes, providing a higher lipid content and reduced amounts of protein [17]. 

**Figure 1:**
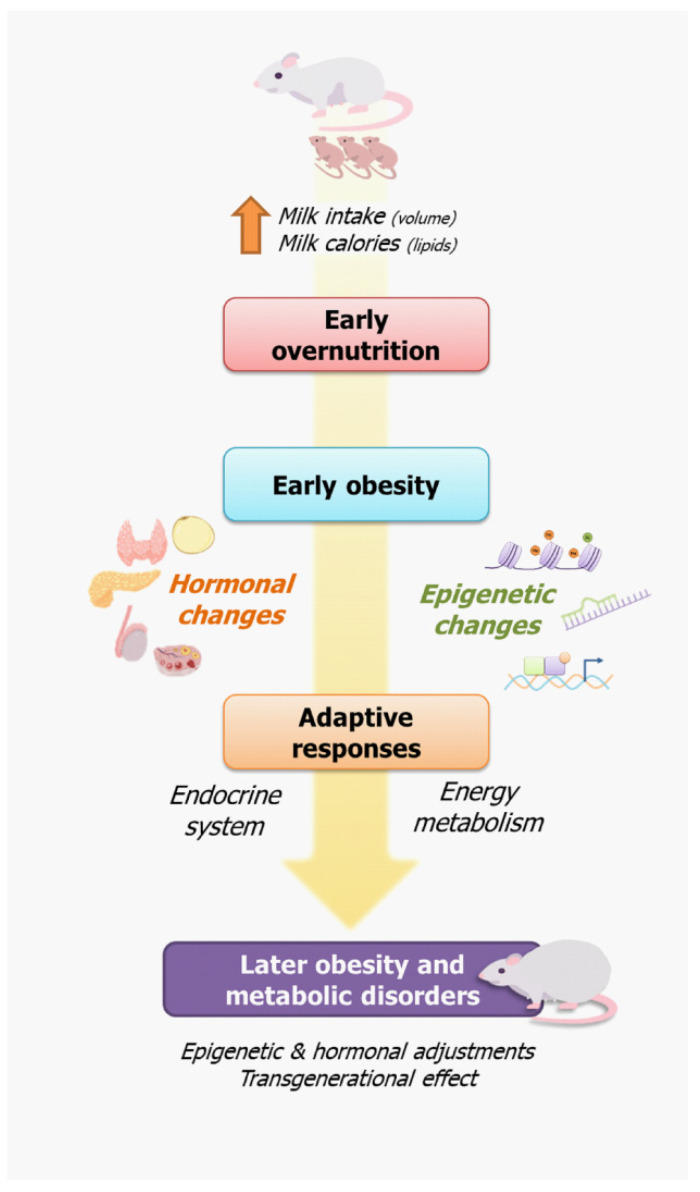
Early overnutrition triggers adaptive responses of the endocrine system and energy metabolism leading to the obesity phenotype of the small litter model. Early overnutrition is a result of increased volume and calorie intake during the lactation period, resulting in early obesity. In response to this early metabolic overload, adaptive responses arise, involving the endocrine system and energy metabolism, which contribute to obesity and metabolic disorders in adult life in offspring raised in small litters.

**Figure 2:**
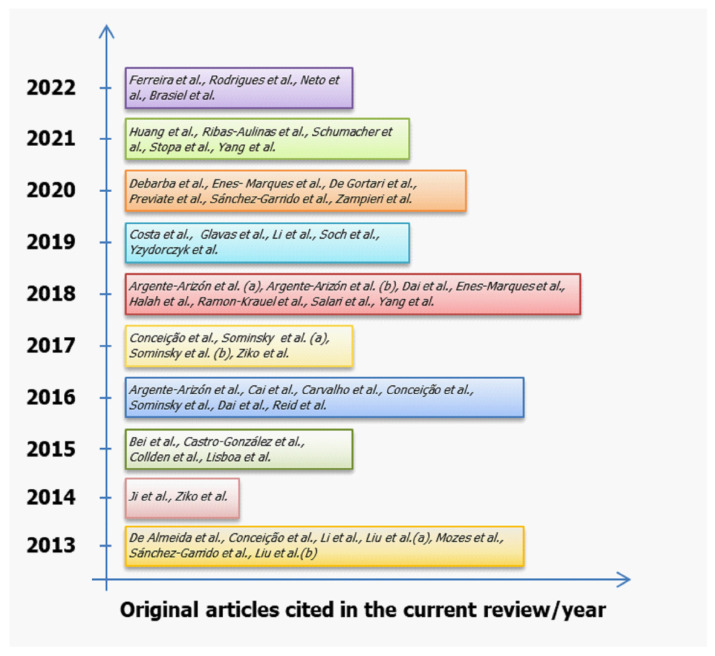
Original articles published per year, since 2013, using the litter size reduction model that was cited in the current review.

Because of this early overfeeding, the SL offspring have an accelerated body weight gain and increased adiposity before weaning, exhibiting a body weight 30% higher than the control offspring at weaning [18]. Some experimental studies have reported persistent hyperphagia, visceral obesity, elevated serum triacylglycerides, increased systolic blood pressure, and hyperinsulinemia in later SL offspring life, findings last reviewed before by Habbout and colleagues [19], featuring a metabolic syndrome phenotype. This is a result of adaptive modifications in response to early overnutrition, including epigenetic and hormonal changes in central and peripheral mechanisms involved in the control of food intake and energy balance in adulthood, which favors the obesity phenotype (Figure 1). This phenotype is reproducible in mice (C57BL/6, Swiss and FVB) and rats (Wistar and Sprague–Dawley); it has been extensively explored in male offspring [15]. Recently, the female response has been investigated, revealing a differentiated phenotype in several aspects. Figure 2 summarizes the 52 original articles cited in the current review, which were published after the Habbout and colleagues review article [19].

### 2.2. Central Dysfunction: Impact on Energy Metabolism and Hormonal Axis

During the lactation period, the hypothalamic circuits that regulate energy homeostasis are still in development and are sensitive to the early hyperinsulinemia and hyperleptinemia exhibited by the SL model [20]. These hormonal changes could be involved in the altered responses to orexigenic and anorexigenic neuropeptides in paraventricular (PVN) hypothalamic neurons [21], favoring hyperphagia.

Then, the metabolic phenotype of SL offspring involves peripheral changes, especially central changes in pathways involved in energy homeostasis control. Many authors have described central resistance to hormones such as leptin [22,23], insulin [24], adiponectin [25], ghrelin [26], and cholecystokinin (CCK) [27]. In addition, there are other central modifications that affect the hormonal axis and contribute to metabolic disorders in adulthood. Adaptive changes in the hypothalamus–pituitary–adrenal (HPA) axis have been reported [28], affecting stress responses; in the hypothalamus–pituitary–thyroid (HPT) axis [29], impacting energy expenditure; and in the reproductive axis [30], causing reproductive disorders. 

Interestingly, several hypothalamic changes occur before weaning, such as leptin resistance at postnatal day (PND) 12 [22], with a downregulation of hypothalamic Ob-Rb at PND 24 [31]. At weaning, the male rat SL offspring show, in the hypothalamic arcuate nucleus (ARC), increased expression of pro-opiomelanocortin (POMC), cocaine and amphetamine regulated transcript (CART), neuropeptide Y (NPY), and ghrelin receptors (GHS-R) [31]. Additionally, this animal model exhibits early alteration of astrocyte morphology at weaning, and it persists until adult life, with increased susceptibility to hypothalamic inflammation [20,32]. This phenotype can affect satiety control and could be due to early hyperinsulinemia and hyperleptinemia [20]. However, this hypothalamic microgliosis does not seem to be the major factor involved in the SL obesity phenotype [33].

In mice, these early changes promoted by neonatal overfeeding could affect neural projections [34] and synaptic transmission [35] in the ARC nucleus of females during adulthood, impacting mechanisms such as satiety control. Supporting the hyperphagic phenotype, SL offspring exhibit higher NPY and lower POMC contents in the ARC [36], suggestive of leptin resistance. Indeed, in adulthood, although their serum leptin and insulin levels are normal, male SL offspring have lower insulin and leptin signaling in the hypothalamus [23] and impairment of leptin-induced satiety [37]. However, leptin resistance is area-specific, since the hypothalamus–pituitary–thyroid axis is still responsive to leptin because the paraventricular (PVN) thyrotropin-releasing hormone (TRH) mRNA levels and T4 serum concentration are increased by exogenous leptin [37].

In addition to hyperphagia, male SL offspring have a food preference for a high-fat diet, an alteration that could be associated with changes in the dopaminergic reward system [36]. This food preference could contribute to a higher susceptibility to metabolic dysfunction in response to high-fat diet exposure [38].

### 2.3. Peripheral Dysfunction

In addition to central dysfunction, SL offspring exhibit several peripheral changes involved in their metabolic profile. The early energy and fat overload promoted by the SL model [17] has been involved in many metabolic disorders by affecting the postnatal development and function of glands such as the pancreas, adrenal, adipose tissue, thyroid, and gonads and metabolic tissues such as liver [17,29,30,39,40,41]. These hormonal changes can act as an imprinting factor or a mechanism of developmental adaptations in metabolic programming models. These hormonal changes can be involved in the adjustments of energy expenditure, stress response, and food intake behavior of the early overfeeding model.

#### 2.3.1. Pancreatic, Insulin, and Glucose Homeostasis 

Early postnatal overnutrition increases the susceptibility to diabetes development [12]. Indeed, SL offspring exhibit many changes in glucose homeostasis throughout life, such as early hyperinsulinemia (PND 10, 13, 15, and 21) and hyperglycemia [12,42,43], which are present [44,45] or not [42] in adulthood. Early pancreatic changes in mice, such as an increased β cell mass (PND 14) [46], reduced islet insulin content, and reduced expression of a marker of islet maturation (pancreatic and duodenal homeobox 1—Pdx1), have been reported, suggesting changes in insulin secretion [46]. Interestingly, it has been reported that isolated islets from adult SL offspring secrete more insulin in the presence of elevated glucose concentrations than islets from normal litter offspring [17,43]. This insulin hypersecretion could be an adaptive and compensatory mechanism, an effort to maintain normal glucose levels, and involves pancreatic changes such as increased glucose transporter 2 (GLUT2) expression [17]. 

In addition to modifications of insulin secretion, adult SL offspring exhibit changes in peripheral insulin signaling, suggestive of insulin resistance. In adult SL male and female offspring, the downregulation of markers of insulin signaling, such as insulin receptor substrate-1 (Irs-1) and glucose transporter 4 (GLUT4) in white adipose tissue and skeletal muscle [44,45], reduces glucose uptake [47] reinforcing the insulin resistance phenotype. Despite these alterations, SL animals exhibit normal glucose tolerance at PND 90 [47], which is disturbed at PND 120 [28], PND 250 [48], and PND 365 [17], suggesting failure of the pancreatic compensatory mechanisms in the long term or under metabolic overload. Indeed, SL mice have accelerated development of diabetes in response to a postweaning high-fat diet compared to a normal litter response [46], highlighting their susceptibility to pancreas dysfunction.

These findings reveal the impact of postnatal overfeeding on offspring susceptibility to diabetes development and the potential mechanisms involved.

#### 2.3.2. Adrenal, Glucocorticoid, Catecholamine, and Stress Response Behaviors

Neonatal overfeeding also affects animal behavior throughout life. The SL offspring exhibits, in adolescence, reduced social play behavior [49] and changes in anxiety-like behavior, which was reduced in the SL from Wistar rats and C57BL/6 mice at PND 60 [50] and increased in the NMRI mice at PND 80 [51]. Differentiated stress-induced corticosterone levels have also been described, increased in the SL from NMRI mice and reduced in the SL from C57BL/6 mice [51]. Modifications in the hypothalamus–pituitary–adrenal (HPA) axis may be involved in this behavior phenotype, causing higher corticosterone levels in response to stress exposure [28].

Indeed, there is a precocious change in the HPA axis of the SL model, exhibiting higher corticosterone levels during early life (PND 14 and 21), together with central changes suggestive of early maturation of the HPA axis [28]. Hypercorticosteronemia is maintained at a young age (PND 56) and throughout adult life (PND 112) [52] in Sprague–Dawley rats, promoting permanent upregulation of the HPA axis and increased peripheral glucocorticoid sensitivity, especially in white adipose tissue in rats [28].

Additionally, in adult SL offspring, a higher adrenal weight with an increased content of corticosterone [28] and catecholamine [39] has been described. In agreement with this finding, there is a higher expression of enzymes of catecholamine synthesis [39]. The SL adrenal gland is more responsive, promoting higher catecholamine secretion in vitro in response to caffeine stimulus [39] and higher corticosterone secretion in vivo in response to adrenocorticotropic hormone (ACTH) stimulus [53]. This hypersecretory profile corroborates the higher serum corticosterone during adult life [52] and increased basal and stress-induced corticosterone secretion in SL offspring [28]. Interestingly, in contrast, the HPA axis is less responsive to fasting and leptin, showing reduced corticosterone levels compared to control animals [37]. These functional changes reveal adaptive mechanisms that contribute to metabolic disorders in the SL model in rats [54].

#### 2.3.3. Adipose Tissue, Leptin, and Hyperphagia

Early postnatal overfeeding promotes increased body weight and adiposity since PND 10 [42], regardless of offspring sex. This phenotype involves mechanisms such as hyperphagia and changes in energy metabolism [19], which are factors targeted by many hormones, such as leptin. This adipocyte-released hormone reflects adiposity, promotes satiety, and increases energy expenditure by central and peripheral actions [55].

The SL offspring of both sexes at PND 10 exhibit higher adiposity and increased serum adipokines, such as leptin and adiponectin [42]. Interestingly, in young adulthood (PND 60), male SL offspring show higher serum leptin [56,57] and lower adiponectin and resistance to central adiponectin action [25], changes that can affect glucose homeostasis.

Neonatal hyperleptinemia per se can be responsible for high adiposity and altered food intake in adult mice [58]. Despite their increased adiposity throughout life, SL offspring have hyperleptinemia at weaning [59] and in young adulthood (PND 60) [56,57], but they show normoleptinemia at PND 180 [29,40]. This normoleptinemia in the presence of increased adiposity could be due to reduced leptin synthesis, since the visceral adipocyte showed lower leptin content [40]. Even in the presence of normal serum leptin, these animals exhibit markers of central leptin resistance [40,60], which explains the increased expression of NPY in the ARC, contributing to the hyperphagic phenotype of this experimental model [60]. Indeed, the adult SL animals present a downregulation of the leptin signaling pathway in the hypothalamus, revealing central leptin resistance [29], which is not observed at weaning.

Interestingly, even in the presence of resistance to the central anorexigenic effect of leptin, SL offspring maintain central sensitivity to activate other central regions, such as the PVN, suggesting that leptin resistance is area-dependent [37]. Additionally, it suggests peripheral resistance to leptin action, as the expected corticosterone suppression is lost in SL offspring after leptin administration [37].

#### 2.3.4. Thyroid Hormones and Energy Expenditure

Postnatal overfeeding modifies energy metabolism, promoting lower energy expenditure at weaning [61] and during adulthood in rodents of both sexes [62]. Hypothalamic and hormonal adjustments, such as leptin resistance in ARC and thyroid hormone levels, contribute to this obesogenic phenotype.

Central and peripheral thyroid hormone action increases the basal metabolism, promoting higher energy expenditure. Even with an adequate hypothalamic–pituitary–thyroid (HPT) axis response to leptin administration, SL offspring exhibit thyroid hormone adjustments throughout life. Interestingly, at PND 21, SL offspring show higher plasma 3,5,3′-triiodothronine (T3) and thyroxine (T4) levels in the presence of increased thyroid-stimulating hormone (TSH) levels [29]. This neonatal hyperthyroidism can act as an imprinting factor, promoting hypothyroidism in adulthood. Indeed, in adulthood, the opposite thyroid profile is observed, with a reduction in T3 and T4 levels, without changes in TSH levels [29]. At this age, SL animals also exhibit reduced hypothalamic TRH content, reduced intrapituitary TSH content, reduced TRH-stimulated TSH release, and increased hypothalamic and pituitary deiodinase (D2) activity [63]. In agreement with this hypothyroidism, the adult SL female offspring have reduced energy expenditure and physical activity [62], and there are changes suggestive of lower thermogenic capacity in adult SL male offspring [64].

Although hypothyroidism occurs, the HPT response to leptin administration is preserved in SL offspring, reinforcing selective leptin resistance in the ARC, the nucleus involved in satiety behavior, but not in the PVN [37].

Brown adipose tissue (BAT), a target of thyroid hormones for thermogenesis, shows reduced responsiveness to cold and lower expression of several transcriptional regulators in young adult male SL offspring [64], suggesting BAT dysfunction. Additionally, at room temperature, adult SL offspring show lower BAT temperature during the light and dark periods [65] and lower levels of uncoupling protein 1 (UCP1) [63,64], reinforcing their reduced thermogenic capacity. Although there are no changes in BAT sympathetic nerve activity [65], the expression of sympathetic beta 3-adrenergic receptor and the response to the sympathetic receptor agonist isoproterenol are decreased in SL offspring [64]. Additionally, BAT dysfunction could involve higher vagus nerve activity [65]. Interestingly, in contrast to the adulthood BAT phenotype, UCP1 expression was elevated in preweaning SL offspring, suggesting increased energy expenditure. This early upregulation may be a temporary adaptive response to both hyperthyroidism and hyperphagia in the experimental model to compensate for excessive energy intake [29,64].

#### 2.3.5. Gonads, Sexual Hormones, and Puberty

Early obesity can affect the endocrine system, modifying the time of puberty. Since the small litter is also a model of early obesity, the effects on sexual parameters have been investigated in male and female offspring. In SL male offspring, serum testosterone levels are higher at PND 10, without changes at weaning, puberty, or adulthood [42]. Although the testicular weight is greater at PND 35 [66], the male SL offspring do not have any changes in the timing of testicular descent or penile gland morphology, suggesting no impact on SL male puberty [42]. However, they exhibit early balanopreputial separation, an external marker of puberty [67]. At PND 90, the SL male offspring shows reduced mass of the epididymis, seminal vesicle, and ventral prostate, without changes in the spermatic parameters [68]. 

On the other hand, female SL offspring exhibit controversial changes at puberty. It has been described as normal [67], early [30], or delayed vaginal opening [68]. Reinforcing the suggestions of early puberty in SL females, they show an early first estrus compared to control animals [30]. At puberty onset, SL female offspring also have higher levels of luteinizing hormone (LH) and follicle-stimulating hormone (FSH) [67] than normal litters. In adulthood, despite the absence of changes in the serum LH and progesterone levels in SL females, they exhibit reduced hypothalamic gonadotropin releasing hormone (GnRH) expression, reduced kisspeptin expression, and lower serum estradiol levels [67]. This disruption in the noradrenergic–kisspeptin–GnRH pathway seems to be involved in the reproductive disorders of SL female offspring [30].

Other reproductive disorders, such as dysregulation of estrous cycles, decreased fertility [30], reduced number of primordial follicles [69], and reduced estradiol plasma concentrations [30], have been reported. Sominsky et al. (2016) also described lower levels of pituitary gonadotropins released at ovulation and altered expression of ovarian markers important for follicular recruitment and survival, which seem to involve increased levels of ovarian leptin and its receptor [70]. Indeed, the postnatal administration of a leptin antagonist to SL female offspring rescues the decline in the primordial follicle pool and abolishes the changes in serum leptin and gonadotropins without changes in body weight [70].

Therefore, in addition to impacting food intake, leptin could be responsible for the changes in the reproductive axis of the SL model [55]. The increased central leptin sensitivity during the peripubertal period seems to be involved in the early activation of the reproductive axis, explaining the higher susceptibility of female SL offspring to reproductive disorders [71] compared to male SL offspring [66]. Leptin is an important permissive signal involved in the timing of pubertal onset. Thus, the increased leptin levels, a reflection of increased adiposity in SL animals, may be related to early puberty and also to other metabolic disorders.

Postnatal overnutrition reduces the fertility index in SL females [34], even in the presence of normal serum progesterone and testosterone levels [30]. Interestingly, female offspring from female SL dams (F2) exhibit increased testosterone levels and reduced estradiol, suggestive of polycystic ovarian syndrome [72].

#### 2.3.6. Liver Metabolism and Dyslipidemia

Early overfeeding affects lipid metabolism, modifying liver homeostasis and promoting dyslipidemia. During the preweaning period, SL mouse offspring have higher serum nonesterified fatty acids (NEFAs) and normal serum triglycerides [73]. This profile is changed during puberty in mice and rats, when the SL offspring exhibits increased serum triglycerides [73], which persists until adult life [61].

In addition to the metabolic adjustments described above, liver dysfunction is another finding of SL offspring and is responsible for dyslipidemia. At weaning, despite the normal hepatic lipid content, the liver from SL offspring exhibits higher expression of lipogenic factors [38,54] and early changes in several clock genes (PND 14) that are maintained until adult life (4–6 months of age), suggestive of circadian rhythm misalignment [74]. Interestingly, the liver also shows early impairment of insulin action (PND 15), which persists until adulthood [73]. These early changes could be involved in the late development of nonalcoholic fatty liver disease (NAFLD) in SL offspring. In adulthood (13 weeks), these animals exhibit increased hepatic triglyceride content [38], areas of inflammatory cell infiltrate, microsteatosis [41], swollen mitochondria, and glycogen deposition [75]. In agreement with this fat accumulation profile, in isolated hepatocytes from SL mice, the incorporation and esterification of free fatty acids into triacylglycerol is higher [73]. Additionally, SL offspring present an imbalance of redox state, with a reduction of oxidative defense and higher oxidative damage and impairment of insulin signaling [41]. This animal model displays early and higher intensity of damage promoted by aging [76] or by a second insult such as a postweaning high-fat diet, showing its susceptibility to liver dysfunction [38].

### 2.4. Epigenetic Changes

Epigenetic changes are the key mechanisms in developmental programming, causing differentiated pattern of gene expression and transgenerational features, without altering the DNA nucleotide sequence [77]. DNA methylation, by the addition of a methyl group on the cytosine of cytosine–guanine dinucleotides (CpG), histone modifications, and microRNA (miRNA) expression, which modify the translation of RNA targets into proteins, are involved in the SL phenotype [73,78,79]. These modifications affect the gene transcription, silencing some genes or promoting overexpression of others, which has an impact on cellular or tissue function. Therefore, these gene changes become adaptive mechanisms in the programming models, modifying cellular signaling, hormonal action, and metabolism. In addition, such epigenetic changes are stably transmitted to the fetus or newborn, favoring the development of metabolic dysfunction in adult offspring. It is possible that some epigenetic changes will be passed on to future generations. At weaning, SL offspring exhibit hypermethylation of the hypothalamic POMC promoter [78], which seems to be involved in the impairment of POMC expression even in the presence of hyperinsulinemia. The insulin receptor (IR) gene promoter is hypermethylated, which is positively correlated with glucose levels [80]. In addition to changes in global hypothalamic methylation in mice [62], changes in pancreatic islet DNA methylation have also been reported, accelerating epigenetic aging in islets and changing the expression of genes involved in insulin secretion, which impairs this function in SL animals [79]. Peripheral changes also contribute to the dysfunction of glucose homeostasis in the SL model. In adult SL offspring, muscular hypermethylation of insulin receptor substrate 1 (IRS1) promotes lower IRS1 expression [45], which could contribute to insulin resistance. In the SL liver, histone modifications may be involved in higher monoacylglycerol acyltransferase 1 (Mogat1) expression, favoring NAFLD development and hepatic insulin resistance [73]. Additionally, there is hepatic miR-221 overexpression, suggesting its involvement in the impairment of the PI3K/AKT pathway in SL offspring [81].

Therefore, some epigenetic changes are involved in the SL offspring phenotype, promoting other adaptive mechanisms, such as hormonal changes. Together, these adjustments favor the increased susceptibility to metabolic disorders in the offspring subjected to early overfeeding. More studies about the epigenome in the SL model are necessary.

### 2.5. Sex-Related Differences

The impact of postnatal overnutrition has been extensively explored in male offspring. Only in recent years has the female phenotype and the comparative response of both offspring to early postnatal overnutrition been explored. These findings have enriched our knowledge about sex-related differences in many developmental programming models [9,82], which could explain the sex differences in the global obesity prevalence and the associated risk of comorbidities.

Sex-related differences were reported regarding the reproductive axis, highlighting the differentiated susceptibility to reproductive dysfunction and time of puberty in the male and female offspring. Postnatal overfeeding increases body weight during the pubertal transition in both sexes [67]. However, differences in the timing of external signs of puberty have been described in male and female offspring [30,67,68]. Interestingly, female SL offspring exhibit higher levels of hormonal markers of puberty, such as LH, FSH, leptin, and insulin, at the time of vaginal opening than normal litter offspring [67]. In males, hormonal changes showed less of a disturbance.

Regarding energy metabolism, early postnatal overnutrition promotes an obesity phenotype in both sexes (PND 10), which can be maintained [18] or abolished in young adulthood (PND 60) [83]. Additionally, in early life (PND 10), the SL male offspring had higher serum insulin, TNF alpha, and HOMA index than the SL female offspring. At the time of puberty onset, leptin levels are higher only in SL females, appearing only in SL males subjected to a combination of postnatal overnutrition and a postweaning high-fat diet [67]. In young adulthood, only SL female offspring exhibit lower BAT thermogenesis during part of the dark phase (at 22 °C), which does not change their whole body energy expenditure [18]. Interestingly, at PND 150, higher adiposity and hyperphagia are observed only in SL male offspring [42], together with higher serum triglycerides and NEFA and signs of hypothalamic gliosis and inflammation [83]. Additionally, despite the early hyperleptinemia in both sexes, postnatal overnutrition promoted hypothalamic changes in NPY and AgRP expression only in the male SL offspring. In female offspring, these central changes did not occur, even in the presence of early hyperleptinemia and adiposity, suggesting sex-dependent differences in the hypothalamic circuits that are sensitive to early overnutrition [84]. These sex-related phenotypes in adulthood suggest an estrogen protective role in female offspring, attenuating metabolic disorders in response to early overnutrition [83] or a contribution of the neonatal testosterone surge [42].

Early overnutrition reduces pituitary growth hormone (GH) mRNA expression in both sexes in response to a high-fat diet. While in the male SL offspring, GH suppression seems to be due to lower GHRH expression, in the female SL offspring, another mechanism is involved, such as reduced pituitary ghrelin action [85]. Indeed, pituitary ghrelin signaling is dysregulated only in female offspring [69].

Recently, our group pointed to the impairment of the gut-brain axis as another contributor to the obesity phenotype in the SL model, whose regulation is also sex-related. In adult SL males, the lower expression of the glucagon-like peptide 1 (GLP-1) receptor in the ARC may impair the satiety role of GLP-1. Despite the paradoxical CCK1-R overexpression in the ARC, the female SL offspring have lower acetate and propionate contents in their feces, which may impair PYY secretion [86]. These different changes may favor hyperphagic behavior in both sexes. Additionally, male and female offspring exhibit dysbiosis (higher abundance of the phylum *Firmicutes*, lower abundance of *Bacteroidetes*), which per se may impair energy metabolism [86], appearing before puberty [87] and being maintained until adult life [86].

The obesity phenotype of the SL female is also of special concern due to its transgenerational role. The offspring from SL female dams, even weaned with 6 pups by the dam, exhibit early obesity and cardiac remodeling in the F2 offspring of both sexes, with cardiac dysfunction only in the F2 male offspring. On the other hand, F2 female offspring show hormonal changes suggestive of polycystic ovarian syndrome, with lower blood estradiol levels and higher testosterone levels [72].

## 3. Final Considerations 

**Figure 3:**
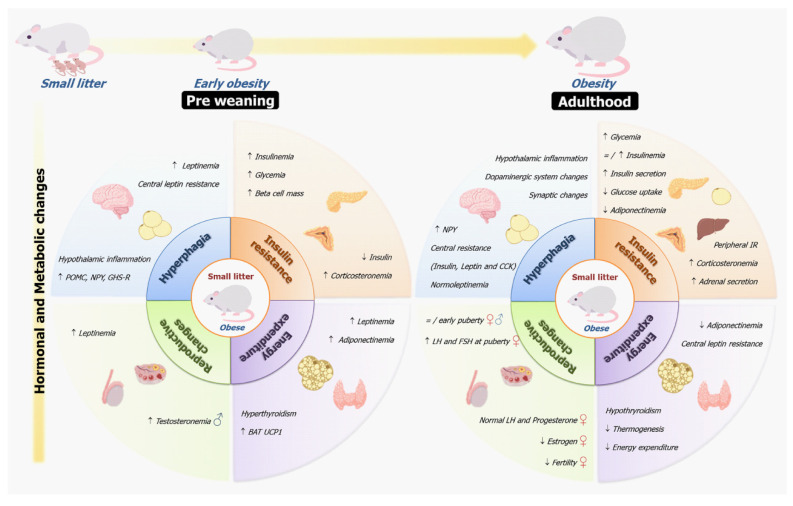
Early and late endocrine and metabolic changes in offspring raised in small litters that explain the development of the obese phenotype. Several hormonal and metabolic changes are involved in hyperphagia, insulin resistance, energy expenditure, and reproductive changes in small litter offspring. There are adaptive responses from preweaning to adulthood that contribute to the obesity phenotype of these animals. Legend: CCK, cholecystokinin; IR, insulin resistance; BAT, brown adipose tissue; UCP1, uncoupled protein 1; POMC, pro-opiomelanocortin; NPY, neuropeptide Y; GHS-R, ghrelin receptors; LH, luteinizing hormone; FSH, follicle-stimulating hormone.

The rodent litter size reduction model is an efficient experimental tool for investigating the impact of early postnatal overnutrition on the development of metabolic diseases. There are early and later adaptive hormonal and metabolic changes described in the literature, which are involved in hyperphagia, energy expenditure changes, insulin resistance, and reproductive changes, featuring the obese phenotype of the small litter model (Figure 3). In programming models, it is difficult to establish a causal relationship of the changes exhibited by the offspring, since several adaptive alterations occurs in different systems at different periods. The overnutrition promoted by increased milk volume and lipid intake can directly affect cell and tissue function, such as that of the pancreas, or indirectly, by hormonal alterations. The early hyperletinemia and hyperinsulinemia in response to overnutrition seem to affect central and peripheral systems, contributing to hyperphagia, changes in the energy expenditure and insulin resistance. The hyperphagia can be due to hypothalamic inflammation and changes in regulatory mechanisms of satiety and energy expenditure, such as the brain–gut axis. Therefore, several complex alterations are involved in the obese phenotype of SL model. These findings demonstrate the importance of the lactation period as a time window susceptible to metabolic disorders and suggest the potential mechanisms involved in the early obesity phenotype and its persistence until adult life. In this review, we described the changes in energy metabolism and its contribution to the obesity phenotype, highlighting the adjustments of the central and endocrine systems as an adaptive mechanism.

Litter size reduction model is an interesting model to study the late impact of early overnutrition. Due to this, the variable “litter size” must be taken into account in the development of any experimental study with animals, regardless of the outcome to be investigated. However, care must be taken to extrapolate the outcomes of this model to human beings, since mothers usually have one baby per pregnancy. Additionally, in humans during breastfeeding, the baby controls the amount of milk intake, self-regulating its energy intake [88]. Therefore, the exclusive breastfeeding, with free demand, does not reflect an early overnutrition in human, which brings several health beneficial effects to the baby during early life [89]. On the other hand, in rats and mice, dams produce about ten to twelve pups that compete for ten viable teats. Thus, the reduction of the number of pups on the third day of lactation to only three pups per mother greatly increases the availability of milk for them. In rodents, this implies a model of overnutrition, as the pup presents early impairment of satiety self-regulation, stopping suckling when its gastrointestinal tract is highly distended [90]. In humans, we can point out that early overnutrition is observed in some situations, for example, due to the inadequate supply of milk from formula artificial dairy products (quality and quantity), a situation in which the baby has less control of volume and energy intake compared to breastfeeding [88]. Early postnatal overnutrition can also occur due to the maternal obesogenic diet, which consequently leads to the production of breast milk with a hypercaloric composition [91]. The persistent obese phenotype of animals raised in a small litter supports its use as an obesity model to investigate anti-obesogenic interventions, especially nutrition, such as fish oil [92], caloric restriction [93], prebiotics [94], calcium [36], *Ilex paraguariensis* (yerba mate) [23], cinnamaldehyde [95], and kefir [96]. These findings show an attenuation of several parameters, revealing that, despite fat accumulation correction, some dysfunctions are not definitely programmed. Therefore, this experimental model that has been extensively explored demonstrates the complexity of obesity programming and the importance of adequate nutrition in early life, avoiding childhood obesity and its impact on health throughout life. Thus, in this review, we highlight the findings of 52 original articles, which increased knowledge about this experimental model since the last review [19]. Epigenetic alterations, hypothalamic inflammation, and the gut–brain axis only recently described are potential mechanisms for disease development in the SL model and also sex-related differences that were not previously investigated.
